# Inhibition of B-cell lymphoma 2 family proteins alters optical redox ratio, mitochondrial polarization, and cell energetics independent of cell state

**DOI:** 10.1117/1.JBO.27.5.056505

**Published:** 2022-05-28

**Authors:** Amani A. Gillette, Rebecca A. DeStefanis, Stephanie L. Pritzl, Dustin A. Deming, Melissa C. Skala

**Affiliations:** a University of Wisconsin, Department of Biomedical Engineering, Madison, Wisconsin, United States; b University of Wisconsin, McArdle Laboratory for Cancer Research, Department of Oncology, Madison, Wisconsin, United States; c University of Wisconsin, Division of Hematology, Oncology and Palliative Care, Department of Medicine, Madison, Wisconsin, United States; d University of Wisconsin Carbone Cancer Center, Madison, Wisconsin, United States; e Morgridge Institute for Research, Madison, Wisconsin, United States

**Keywords:** optical redox ratio, autofluorescence, mitochondria, nicotinamide adenine dinucleotide hydrogen, flavin adenine dinucleotide

## Abstract

**Significance:**

The optical redox ratio (ORR) [autofluorescence intensity of the reduced form of nicotinamide adenine dinucleotide (phosphate) (NAD(P)H)/flavin adenine dinucleotide (FAD)] provides a label-free method to quantify cellular metabolism. However, it is unclear whether changes in the ORR with B-cell lymphoma 2 (Bcl-2) family protein inhibition are due to metabolic stress alone or compromised cell viability.

**Aim:**

Determine whether ABT-263 (navitoclax, Bcl-2 family inhibitor) changes the ORR due to changes in mitochondrial function that are independent of changes in cell viability.

**Approach:**

SW48 colon cancer cells were used to investigate changes in ORR, mitochondrial membrane potential, oxygen consumption rates, and cell state (cell growth, viability, proliferation, apoptosis, autophagy, and senescence) with ABT-263, TAK-228 [sapanisertib, mammalian target of rapamycin complex 1/2 (mTORC 1/2) inhibitor], and their combination at 24 h.

**Results:**

Changes in the ORR with Bcl-2 inhibition are driven by increases in both NAD(P)H and FAD autofluorescence, corresponding with increased basal metabolic rate and increased mitochondrial polarization. ABT-263 treatment does not change cell viability or induce autophagy but does induce a senescent phenotype. The metabolic changes seen with ABT-263 treatment are mitigated by combination with mTORC1/2 inhibition.

**Conclusions:**

The ORR is sensitive to increases in mitochondrial polarization, energetic state, and cell senescence, which can change independently from cell viability.

## 1 Introduction

Multiphoton autofluorescence imaging provides an attractive label-free approach to test new drugs and drug combinations for cancer treatment as it is non-destructive and can provide high-resolution single-cell information. This technique detects NAD(P)H and flavin adenine dinucleotide (FAD) which perform the functions of electron donor and acceptor, respectively, across multiple metabolic reactions within cells.^1^^,^^2^ The fluorescent properties of the reduced forms of nicotinamide adenine dinucleotide (NADH) and reduced nicotinamide adenine dinucleotide phosphate (NADPH) overlap and are jointly referred to as NAD(P)H. However, it is important to note that they play different roles in metabolic pathways, and while the NADH/NAD+ ratio is generally less oxidized compared to the NADPH/NADP+ ratio, the two are coupled. This suggests that changes in the combined NAD(P)H autofluorescence can be used to monitor for changes in the reduced state of the individual cofactors.^3^^–^^5^ Multiphoton microscopy of NAD(P)H and FAD can be used to image the optical redox ratio (ORR) [intensity of NAD(P)H divided by intensity of FAD], which provides a label-free measurement of the oxidation-reduction state of a cell.^6^^–^^9^ Optical redox imaging is advantageous compared to standard measurements of metabolism and cell state because it is amenable to repeated measures of single cells within intact, unperturbed systems.^10^^–^^13^ Previous studies have shown that the ORR is correlated with oxygen consumption rate (OCR) as measured by a Seahorse assay,^14^^,^^15^ as well as with NAD+/NADH ratios as measured by liquid chromatography/mass spectrometry.^16^ Although several prior studies have confirmed that the ORR can reliably predict drug efficacy *in vitro* and *in vivo*,^17^^–^^21^ there are multiple factors that contribute to changes in the ORR within a cell including temperature, pH, cell density, substrate availability, and mitochondrial function.^11^^,^^12^^,^^22^^–^^25^ Therefore, the ORR can be more difficult to interpret than standard measurements of metabolism and cell state.

For example, previous multiphoton imaging studies indicate that the ORR changes with ABT-263 [navitoclax, B-cell lymphoma 2 (Bcl-2), and B-cell lymphoma-extra large (Bcl-xL) inhibitor] treatment in patient-derived neuroendocrine tumor organoids.^26^ However, it was unclear whether changes in the ORR with ABT-263 treatment were due to metabolic stress alone or compromised cell viability. Importantly, the Bcl-2 family of proteins are located on the outer mitochondrial membrane and canonically, they control mitochondrial outer membrane permeabilization (MOMP), which is a key step in apoptotic cell death.^27^^–^^29^ However, it has also become clear that most Bcl-2 family proteins also play roles in cellular processes unrelated to apoptosis, including metabolism, proliferation, autophagy, and senescence.^30^ These processes all affect the oxidation–reduction state of the cell and thus, the ORR.^8^^,^^13^^,^^31^ As a monotherapy, ABT-263 has shown limited efficacy in clinical trials but is currently under investigation as a combination treatment with mammalian target of rapamycin complex 1/2 (mTORC 1/2) inhibitors.^28^^,^^32^^–^^34^ Synergism between mTORC 1/2 inhibitors and Bcl-2 inhibitors has been observed in pre-clinical studies of small cell lung cancer and leukemia.^35^^,^^36^ Due to the complex set of biological functions that the Bcl-2 family of proteins is known to influence, it is of interest to investigate whether ABT-263 alters the ORR of cancer cells due to metabolic shifts that are independent of cell state.

Since Bcl-2 inhibition and mTORC 1/2 inhibition are synergistic for cancer treatment, we investigated ABT-263 alone and in combination with an mTORC 1/2 inhibitor, TAK-228 (sapanisertib).^35^^,^^36^ We hypothesize that changes in the ORR with ABT-263 treatment may be due to changes in cell state that are independent of changes in cell viability. To test this hypothesis, we measured changes in ORR, mitochondrial membrane potential, OCR, and cell state (cell growth, viability, proliferation, apoptosis, autophagy, and senescence) in SW48 colon cancer cells after treatment with ABT-263, TAK-228, and their combination at 24 h. To our knowledge, these are the first studies to measure changes in ORR alongside autophagy, senescence, and mitochondrial membrane potential. These experiments will determine the sensitivity of the ORR to inhibition of Bcl-2 family proteins and provide context for ORR measurements with respect to standard measurements of metabolism and cell state.

## 2 Methods

### 2.1 Cell Culture and Treatment

SW48 colon cancer cells (ATCC – CCL-231) were maintained in RPMI-1640 (Gibco) supplemented with 10% fetal bovine serum (FBS) (#TMS-013-B, Millipore) and 1% penicillin-streptomycin (#15070, Gibco). Cells were grown at 37°C in 5% ${\mathrm{CO}}_{2}$ and passaged at 70% confluency. For all experiments, a treatment of 250-nM ABT-263 (ApexBio cat# A3007), 10-nM TAK-228 (LC Laboratories cat.# I-3344), or the combination was used. These doses are based on physiologically relevant blood serum levels of ABT-263^37^^,^^38^ and on a WST-1 dose response curve for TAK-228, where 10 nM was shown to decrease proliferation of cells by >50% compared to control.^39^ Cells were exposed to treatment for 24 h.

### 2.2 Tetramethylrhodamine, Ethyl Ester, Perchlorate Imaging

For all imaging experiments, $1.8\times 10^{5}$ cells were plated 48 h prior to imaging in 2-ml media on 35-mm glass-bottom dishes (#P35G-1.5-14-C, MatTek Corp). Treatments were started 24 h after seeding (10-nM TAK-228, 250-nM ABT-263). On the day of imaging, 10-nM tetramethylrhodamine, ethyl ester, perchlorate (TMRE) was added to the dishes, which were then incubated at 37°C in 5% ${\mathrm{CO}}_{2}$ for 1 h. No washes were performed, and cells were then imaged at six different fields of view (FOV) in two biological replicates for a total of 12 FOVs per condition and 170 to 230 cells imaged per condition. Non-quenching mode in these cells was validated with carbonyl cyanide 4-(trifluoromethoxy)phenylhydrazone (FCCP) treatment in our previous paper.^40^

Fluorescence intensity (FI) images were acquired using a custom-built multiphoton fluorescence lifetime system (Bruker fluorescence microscopy), with a 100× oil-immersion objective [1.45 numerical aperture (NA), Nikon] and an inverted microscope (TiE, Nikon). An ultrafast laser (Insight DS+, Spectral Physics) was tuned to 750 nm for two-photon excitation of NAD(P)H and a fixed line at 1040 nm for simultaneous two-photon excitation of TMRE. NAD(P)H images were acquired to aid with single-cell segmentation of the TMRE signal. A 440/80-nm bandpass filter was used to collect NAD(P)H fluorescence emission, and a 575-nm long-pass mirror with a 645/50-nm filter was used to collect TMRE emission. An optical zoom of 2 and a pixel dwell time of 2  μs collected 1024×1024  pixel images, averaging four frames. A GaAsP photomultiplier tubes (H7422P-40, Hamamatsu Photonics) detected emitted photons.

The semi-automated image analysis code was created using standard and customized modules within CellProfiler (v.4.0.7) as previously described.^40^ Briefly, the NADH(P)H image was adjusted to a custom maximal intensity to increase contrast for future segmentation steps. The TMRE image was then smoothed using a circular average filter and thresholded using a global minimum cross-entropy with a threshold correction factor of 2 and no smoothing. Next, the rescaled NAD(P)H image was used to manually segment the nuclei of each cell, cells were then identified by propagating out from the nuclei, a minimum cross-entropy threshold was used to improve the propagation. The nucleus was then subtracted from the cell to identify the cell cytoplasm. The cytoplasm was then masked to isolate only the mitochondrial signal based on the TMRE threshold that identifies the bulk mitochondrial component on a per cell level. Finally, the intensity values of TMRE were measured for each of the different cellular compartments, nuclei, cells, cytoplasm, and mitochondria for each cell, between 177 and 225 cells were analyzed per condition.

The values exported from CellProfiler for the TMRE intensity in the nucleus and the mitochondria were then used in Nernst equation Eq. (1) to calculate the mitochondrial membrane potential for each cell,

$$\Delta \Psi =-RT*\log(\frac{{\mathrm{FI}}_{m}}{{\mathrm{FI}}_{n}}),$$ (1)

where RT=61.5 at 37°C, and ${\mathrm{FI}}_{m}$ = fluorescence intensity of mitochondria, ${\mathrm{FI}}_{n}$ = fluorescence intensity of the nucleus.^41^ This equation is based on previous work that showed TMRE accumulation is similar in the nucleus and cytoplasm.^41^^,^^42^ The nuclear TMRE signal was used to minimize potential corruption of the cytoplasmic TMRE signal from mitochondrial TMRE contributions.

### 2.3 Optical Redox Imaging

Optical redox imaging was performed on a custom inverted multiphoton microscope (Bruker fluorescence microscopy), as described above. Four independent FOVs were acquired per condition in photon counting mode using time correlated single photon counting electronics (SPC-150, Becker and Hickl) and a GaAsP photomultiplier tube (H7422P-40, Hamamatsu). NAD(P)H and FAD images were acquired sequentially for the same FOV using a 750-nm excitation wavelength and a 440/80-nm emission filter for NAD(P)H, and an 890-nm excitation wavelength and a 550/100-nm emission filter for FAD. A pixel dwell time of 4.8  μs was used to acquire 512×512  pixel images over 60 s total integration time. The photon count rates were maintained at $2\times 10^{5}$ to $6\times 10^{5}\text{ photons}/s$ to ensure adequate photon observations and no photobleaching.

For data collected in photon counting mode, at each pixel, a bin of 3×3 was used and two-component NAD(P)H and FAD fluorescence decay curves were separately integrated to generate the intensity of each fluorophore using SPCImage software (Becker and Hickl). The intensity of NAD(P)H $\left[I_{\mathrm{NAD}(P)H}\right]$ was then divided by the intensity of FAD ($I_{\mathrm{FAD}}$) for each pixel to calculate the ORR,

$$\mathrm{ORR}=\frac{I_{\mathrm{NAD}(P)H}}{I_{\mathrm{FAD}}}.$$ (2)

An automated cell segmentation pipeline was created in CellProfiler and applied to NAD(P)H intensity images as previously described.^43^ Briefly, pixels belonging to nuclear regions were manually identified and the resulting round objects were stored as a mask. Cells were identified by propagating out from each nucleus, an Otsu Global threshold was used to improve the propagation and prevent it from continuing into background pixels. Cell cytoplasm was defined as the cell border minus the nuclei. Values for intensity of NAD(P)H and FAD as well as the ORR were measured for each cell cytoplasm. For each variable, values for all cells within a treatment group were pooled together, 430 to 730 cells were analyzed per condition.

### 2.4 Immunofluorescence

After optical redox imaging, cells were fixed with 2% paraformaldehyde for 15min and then washed once with PBS before storage in PBS overnight. The next day immunofluorescence staining was performed as previously described.^44^ Conjugated antibodies against Ki67 and CC3 (11882S-488 conjugate, and 8172S-594 conjugate, Cell Signaling Technology, Danvers, Massachusetts) both at a dilution of 1:50 were applied before coverslips were mounted onto slides with Prolong Gold DAPI mounting media (P36931, Invitrogen, Carlsbad, California) and sealed with clear nail polish. After 24 h stored at 4°C in a dark box, the slides were imaged on a Nikon Eclipse Ti2 inverted widefield fluorescence microscope using a 20× air objective (0.75 NA), with standard Nikon filters for DAPI, FITC, and Texas Red [DAPI: ex: 375/28  nm, em: 460/60  nm; FITC: ex: 480/30  nm, em: 535/45  nm; Texas Red: ex: 560/40  nm, and em: 630/60  nm). For analysis, six FOVs were acquired from two separate biological replicates for a total of 12 FOVs analyzed per condition. Co-localization analysis was performed using a previously described CellProfiler pipeline^26^ that identifies DAPI, Ki67, and CC3 objects. The number of DAPI objects that overlapped with Ki67 or CC3 positive stain were counted to calculate the percent of total cells positive for Ki67 or CC3.

### 2.5 Extracellular Flux Measurements for Metabolic Profiling

The OCR and the extracellular acidification rate (ECAR) were measured with a Seahorse Extracellular Flux Analyzer XFe96 (Agilent). Cells were plated at a density of $2\times 10^{5}\text{ cells}/\text{well}$ 48 h prior to assay, 24 h after plating treatments were started (10-nM TAK-228, 250 nM ABT-263). The assay medium consisted of pH adjusted RPMI (Agilent) supplemented with glucose (25 mM) and glutamine (4 mM). Cells were washed twice with assay media and incubated for 1 h in a 37°C non-${\mathrm{CO}}_{2}$ incubator prior to assay start.

The Seahorse XF mito stress test was used to measure the OCR and ECAR according to the manufacturer’s instructions. In brief, the test uses a sequence of mitochondrial inhibitors: oligomycin (2  μM), FCCP (0.25  μM), and rotenone/antimycin A (0.5  μM/0.5  μM). Baseline OCR and ECAR were monitored for three cycles of 6 min followed by sequential inhibitor injections with three cycles of OCR and ECAR measurements following each injection. Calculations of OCR and ECAR were performed by Seahorse XF-mito stress assay report generator exported from Wave 2.6.1 software. The OCR and the ECAR were normalized for deoxyribonucleic acid (DNA) per well as quantified by crystal violet absorbance. Basal respiration was calculated by subtracting the post-rotenone + antimycin A average from the pre-treatment average for each well. After completion of assay, plates were fixed with 1% glutaraldehyde for 15 min at room temperature, plates were then washed two times with deionized (DI) water and allowed to air dry for 20 min. Then 100  μl of 0.1% crystal violet in DI water was added to each well and incubated on a rocker at room temperature for 20 min. Finally, the plate was washed twice in DI water and allowed to air dry overnight. The next day 100  μl of 10% acetic acid in DI water was added to each well, and absorbance was measured at 590 nm.

### 2.6 Viability Assays

The SW48 cells were plated at $5\times 10^{4}\text{ cells}/\text{well}$ in 500  μl in a clear walled plastic 24 well plate. After allowing the cells to settle for 48 h, four wells were collected for a baseline cell count as described below. Cells were treated with either control, 250-nM ABT-263, 10-nM TAK-228, or the combination and incubated for 24 h at 37°C and 5% ${\mathrm{CO}}_{2}$. Treated cells were then trypsinized and total cell number determined using a hemocytometer. Cell counts were performed on three individual wells and two separate 24 well plates for each condition. After counting, the average of the 48-h baseline cell count was subtracted from the 24-h post-treatment cell count. The values were then divided by the average of the control condition and multiplied by 100 to calculate the cell count as a percent of control.

SW48 cells were plated at $9\times 10^{3}\text{ cells}/\text{well}$ in 100  μl in a clear walled plastic 96 well plate. After allowing the cells to adhere for 48 h, cells were treated with 250-nM ABT-263 and 10 nM TAK-228. After a 48 h incubation, the media containing drug were aspirated and the WST-1 reagent made in fresh media was added per the manufacturer’s protocol (CELLPRO-RO, Roche). The plate was wrapped in tin foil and incubated for 0.5 to 4 h at 37°C and 5% ${\mathrm{CO}}_{2}$ with reads taken every 0.5 h at both 450 and 650 nm until the reads in the control wells reached a range close to 0.8 at the 450-nm wavelength. For analysis, the background phenol red signal from media (650 nm) was subtracted from the WST-1 signal (450 nm) for each well (16 wells per treatment condition). The control wells (no cells, 16 wells) were then averaged and this background level was subtracted from each treatment well. Finally, wells within a treatment condition were averaged.

### 2.7 CytoID Autophagy Assay

SW48 cells were plated at $8\times 10^{4}\text{ cells}/\text{well}$ in 100-μl per well, in glass bottom black-walled 96 well plate (CellVis) and allowed to attach for 24 h. Assay was adapted from CytoID detection kit product manual (Enzo Life Sciences). Briefly, half the wells were treated with 50-μM chloroquine added 1 hr before other treatments. Then individual wells were treated with 100-nM rapamycin, 250-nM ABT-263, 10-nM TAK-228, TAK-228+ABT-263, or a DMSO control (five wells per condition). Plates were incubated for 24 h at 37°C and 5% ${\mathrm{CO}}_{2}$. The next day, the assay buffer (5-ml 10× assay buffer + 45-ml DI $H_{2}O$, supplemented with 5% FBS) and CytoID detection solution (10-μl Cyto-ID green detection reagent, 10-μl Hoechst 33342 nuclear stain into 10-ml 1× assay buffer) were made as per protocol. Wells were washed with 100-μl assay buffer, before addition of 100-μl CytoID Detection solution, plates were incubated at 37°C and 5% ${\mathrm{CO}}_{2}$ for 30 min, then washed with 200-μl 1× assay buffer, before a final 100-μl 1× assay buffer was added for the read. Plate was read on a fluorescence microplate reader (CytoID: ex: 480 nm, em: 530 nm; Hoechst: ex: 340 nm, and em: 480 nm). CytoID signal was normalized to the Hoechst signal in each well to account for altered cell numbers.

### 2.8 β-galactosidase Senescence Assay

Cells plated at $1.8\times 10^{5}\text{ cells}/35\text{-}\mathrm{mm}$ glass bottom dish and allowed to attach overnight. Treatment started the next day with 250-nM ABT-263 and 10 nM TAK-228 for 24 h (three dishes per condition). Senescence was determined using the Senescence Cells Histochemical Staining Kit (Sigma-Aldrich, CS0030). Briefly, 1× fixation buffer, and 1× PBS were made fresh from 10× stocks and ultrapure water on day of experiment. Staining mixture was made immediately before use by mixing 1 ml of the 10× staining solution, 125  μl of reagent B, 125  μl of reagent C, 250  μl of X-gal Solution, 8.5-ml ultrapure water, and then filtered through a 0.2-μm filter. The media were carefully aspirated off all dishes and the cells were washed twice with 1× PBS. After washing, 1.5 ml of 1× fixation buffer was added to each well and incubated at room temperature for 6 and 7 min, fixing was followed by three additional 1× PBS washes. Finally, 1 ml of the prepared staining mixture was added to each well and each plate was sealed with parafilm. Dishes were then incubated at 37°C with no ${\mathrm{CO}}_{2}$ overnight. The next day dishes were imaged under a bright-field microscope (Nikon Eclipse Ti2). The percentage of senescence (X-gal) positive cells was quantified using ImageJ software.

### 2.9 Statistical Analysis

Differences in variables between treatment and control cells were tested using a one-way analysis of variance of control versus treatment, a Dunnett’s multiple comparisons test with a single pooled variance was used to explore differences between multiple groups, all p-values<0.01 were defined as significant.

## 3 Results and Discussion

### 3.1 Optical Redox Ratio Decreases with ABT-263, TAK-228, and their Combination

**Fig. 1:**
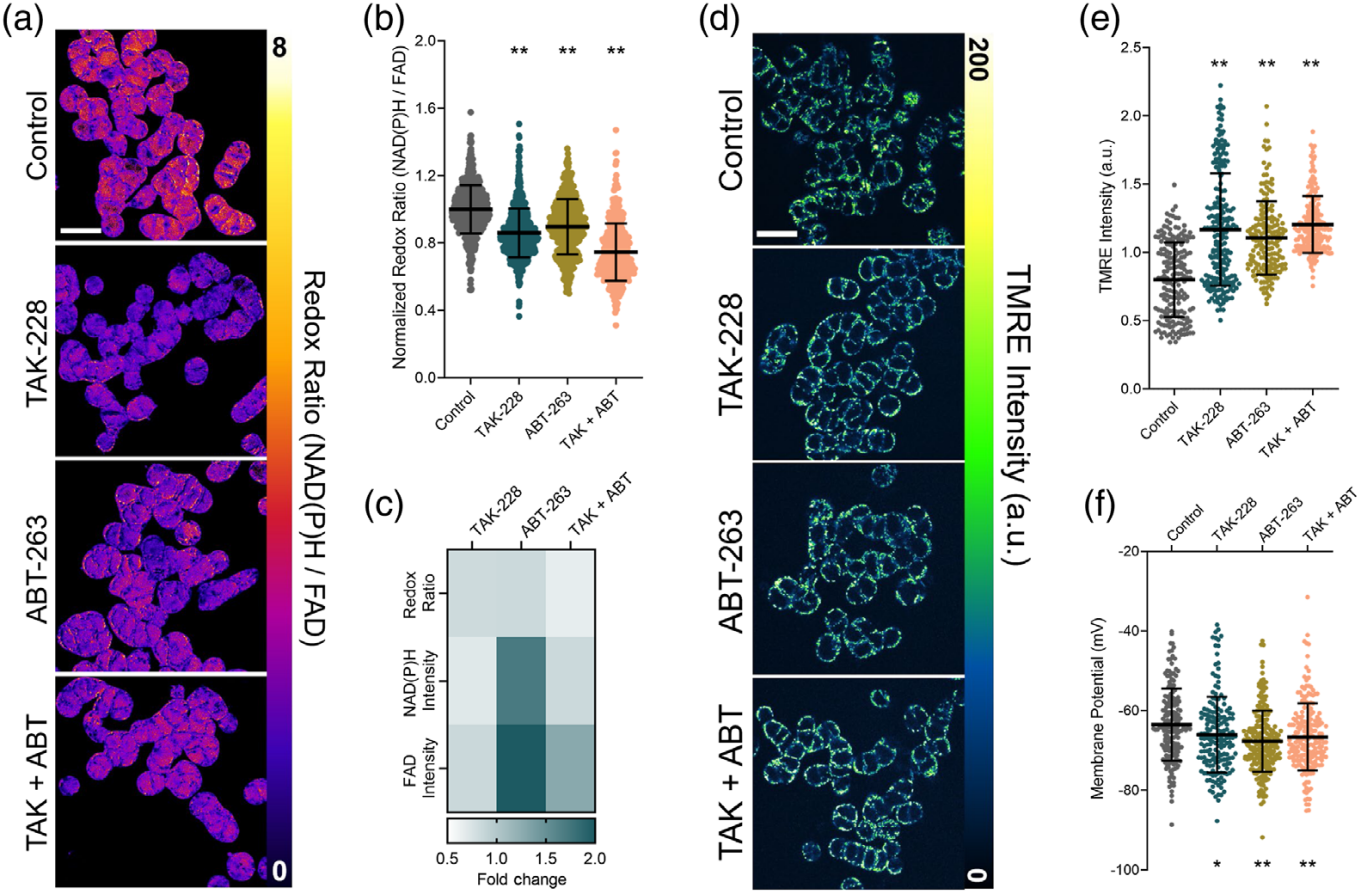
ORR decreases, and mitochondrial polarization increases with ABT-263, TAK-228, and their combination. (a) Representative images of ORR for all treatment conditions in SW48 cells (scale bar=25  μm). (b) Cell-level ORR normalized to control for each treatment, data points plotted individually with the mean and standard deviation overlayed. 430 to 730  cells/condition. (c) Heatmap of the fold-change relative to the control for autofluorescence variables. (d) Representative images of TMRE false colored to show intensity in SW48 cells (scale bar=25  μm). (e) Cell-level TMRE intensity for each treatment, data points plotted individually with the mean and standard deviation overlayed. 177 to 225  cells/condition. (f) Cell-level mitochondrial membrane potential for each treatment, calculated from Eq. (1). (*p<0.01) and (**p<0.005).

Measurements of metabolism were acquired at an early time-point in treatment (24 h post-treatment) to capture changes in cell state that often precede standard drug response measurements.^45^ Representative ORR images of SW48 cells after 24 h of treatment indicate a decrease in ORR for ABT-263, TAK-228, and their combination [Fig. 1(a)]. Published work has shown that a decrease in ORR often predicts later treatment response.^19^^,^^46^ At the single-cell level, the ORR significantly decreases for all treatment conditions relative to control [Fig. 1(b)]. This decrease was greatest with the combination therapy, down to 0.75 of the control, which is consistent with prior studies that show synergy with a combination treatment of ABT-263 and TAK-228.^35^^,^^36^ The decrease in ORR with ABT-263 treatment alone, down to 0.89 of the control, is driven by a near two-fold increase in both the NAD(P)H and FAD intensities compared to control, 1.70 and 1.99, respectively [Fig. 1(c)]. This increase in intensity corresponds with an increase in the concentration of NAD(P)H and FAD present in the cells after ABT-263 treatment that is not seen in the other treatment conditions.^47^ Importantly, these decreases in ORR with Bcl-2 and mTORC1/2 are consistent with prior our studies in different cell lines and/or primary patient samples.^26^

### 3.2 Mitochondrial Polarization Increases with ABT-263, TAK-228, and their Combination

Bcl-2 family proteins, the target of ABT-263, help regulate mitochondrial membrane integrity. Therefore, to further investigate the increased concentration of NAD(P)H and FAD observed in the ABT-263 treatment condition, we imaged treatment-induced changes in mitochondrial membrane potential using TMRE staining in a non-quenching mode. Representative TMRE images show uniform staining, and consistent mitochondrial localization in the cytoplasmic space of the SW48 cells [Fig 1(d)]. There is a significant increase in TMRE intensity with all treatments compared to control [Fig. 1(e)], which reflects an increase in the polarization of the mitochondria.^48^ The Nernst equation Eq. (1) provides a calibrated quantitative measurement of mitochondrial membrane potential and confirms that the mitochondria are more polarized in SW48 cells after all treatments compared to control [Fig. 1(f)]. These increases in TMRE intensity and mitochondrial polarization [Figs. 1(d)–1(f)] are consistent with the more oxidized redox state post-treatment measured with the ORR [Figs. 1(a)–1(c)], in agreement with prior biochemical measurements of mitochondrial redox state and membrane potential^24^ Furthermore, the increase in mitochondrial polarization indicates that there is not an increase in mitophagy, which is driven by membrane depolarization.^49^^–^^52^ However, the change in mitochondrial membrane potential does not completely account for the significant increase in the concentrations of NAD(P)H and FAD observed with ORR imaging.

### 3.3 ABT-263 Increases the Energetic State of SW48 Cells

**Fig. 2:**
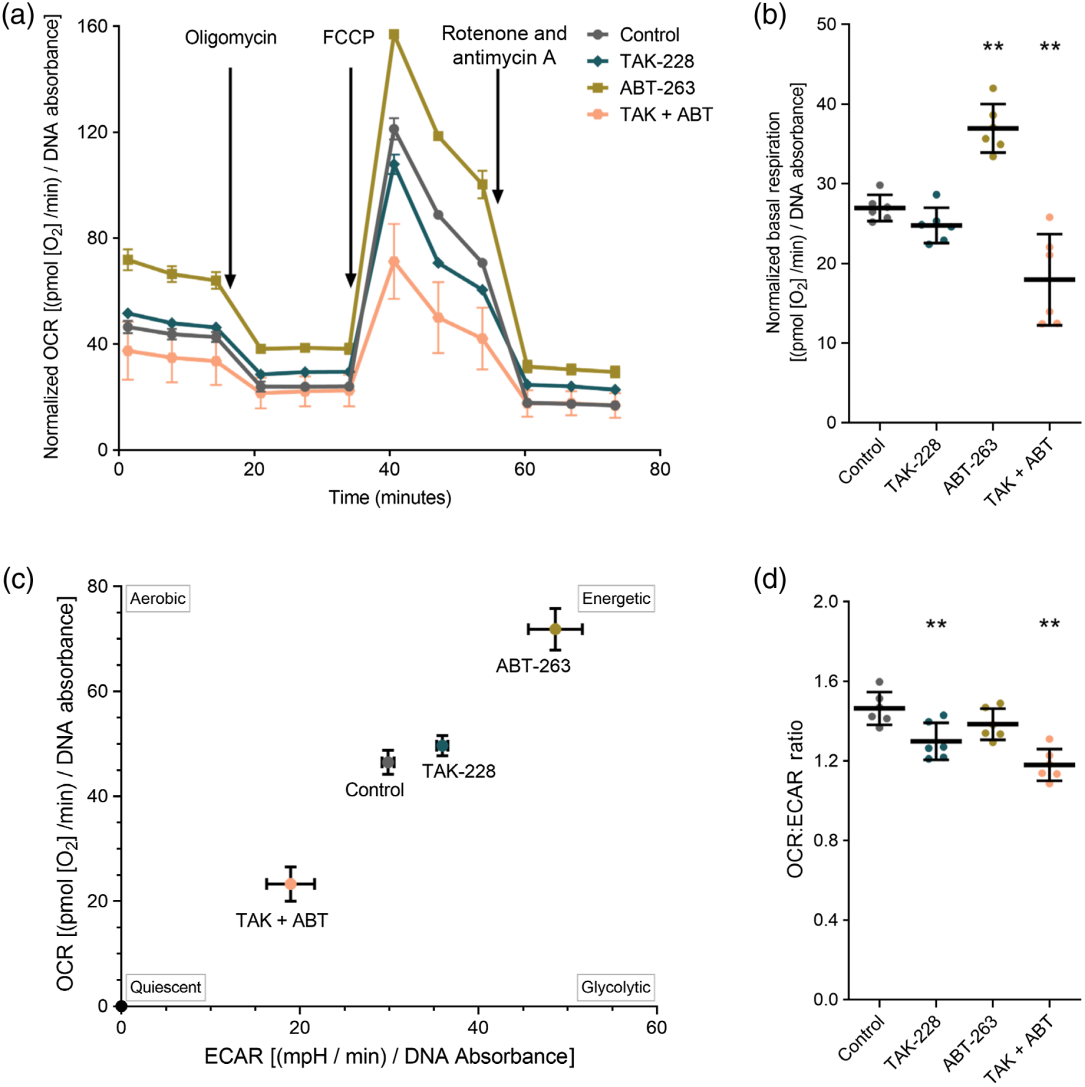
ABT-263 increases the energetic state of SW48 cells. (a) OCR normalized to total DNA for control, ABT-263, TAK-228, and their combination (TAK + ABT) treatment. Time points for metabolic inhibitor treatments are indicated with arrows. (b) Basal respiration normalized to total DNA was determined from the traces in (a) by calculating the pre-treatment average minus the post-rotenone + antimycin A average for each condition, data points plotted individually with the mean and standard deviation overlayed (**p<0.005). (c) OCR versus ECAR visualizes shifts in metabolism from the control condition, characterized by the quadrants of the plot as aerobic, quiescent, energetic, or glycolytic. (d) The OCR to ECAR ratio measures relative use of oxidative versus glycolytic energy production pathways, data points plotted individually with the mean and standard deviation overlayed. (**p<0.005).

Another method to measure cell metabolism, and especially mitochondrial metabolism, is the Seahorse Mito Stress test which measures altered oxygen consumption between treatment groups using the normalized OCR.^53^ Additionally, ABT-263 treatment increases oxygen consumption while TAK-228 and combination treatment decrease oxygen consumption compared to control [Fig. 2(a)]. The basal mitochondrial respiration (BMR) is a measure of the energetic demand of the cell under baseline conditions. Calculating the BMR for each treatment shows that ABT-263 treatment alone significantly increases BMR, TAK-228 does not change BMR, and combination treatment significantly decreases BMR [Fig. 2(b)]. This data show that ABT-263 treatment increases the oxygen consumption of SW48 cells. The OCR versus ECAR plot visualizes shifts in metabolism with treatment compared to control. ABT-263 treated cells shift toward the upper right quadrant of this plot, indicating a more energetic state compared to control, while combination treatment shifts cells to a quiescent phenotype [Fig. 2(c)]. This provides evidence that the ABT-263 treated cells have increased OCR and increased ECAR, which reflect increases in both glycolytic and oxidative energy production pathways. Finally, the OCR to ECAR ratio measures relative use of oxidative versus glycolytic energy production pathways. The significant decrease in OCR to ECAR ratio with TAK-228 and combination treatment suggests that these conditions promote increased use of glycolytic pathways compared to control [Fig. 2(d)].^50^ There is no significant change in the OCR to ECAR ratio after ABT-263 treatment [Fig. 2(d)] even though this treatment creates a more energetic phenotype, indicating similar increases in both OCR and ECAR with ABT-263 treatment [Fig. 2(c)]. Interestingly, the increased energetic state seen in the mito stress test, with increases in glycolytic and oxidative energy metabolism after ABT-263 treatment, is consistent with the increased concentrations of both NAD(P)H and FAD measured in Fig. 1(c). However, increases in energetic state measured by Seahorse and autofluorescence do not directly report on the cell state induced by ABT-263 treatment.

### 3.4 ABT-263 Treatment Does Not Change Cell Viability or Induce Autophagy But Does Induce a Senescent Phenotype

**Fig. 3:**
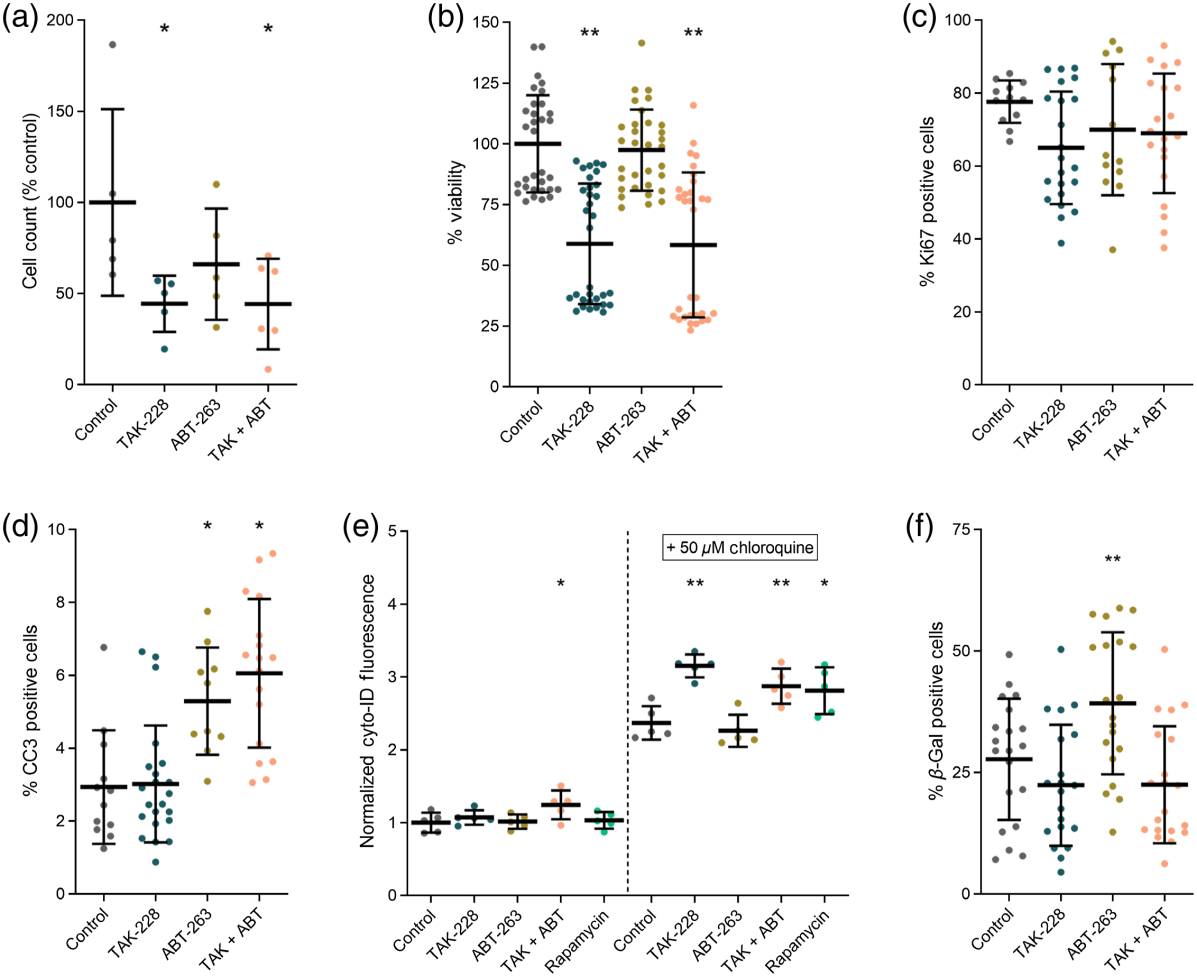
Measurements of cell fate at 24 h post-treatment in SW48 cells. (a) Cell counts [((24 h post-treatment − pre-treatment)/control)*100] show that ABT-263 alone does not change cell growth, while TAK-228 and combination (TAK + ABT) treatments both decrease cell growth compared to control. (b) Percent viability measurements with a WST-1 plate-based assay indicate that ABT-263 treatment alone does not decrease cell viability while TAK-228 and combination treatments both decrease cell viability compared to control. (c) and (d) Immunofluorescence staining for Ki67 and CC3 markers for cell proliferation and cell death respectively. No significant changes were seen in cell proliferation across all treatment conditions, while ABT-263 and combination treatments caused a small but significant increase in CC3 positive cells. (e) Autophagy measurements of fluorescence accumulation in autophagosomes (Cyto-ID plate-based assay) show no change with the positive control (rapamycin), indicating that signal is below reliable detection levels. Chloroquine prevents the degradation of autophagosomes and leads to an increase in signal with more reliable assay results. With chloroquine treatment, increases in autophagy are seen for all conditions except ABT-263 treatment. (f) β-galactosidase, a marker for senescence, increases for ABT-263 treatment only. All plots show data points plotted individually with the mean and standard deviation overlayed (*p<0.01) (**p<0.005).

To confirm that cell viability was not significantly affected by ABT-263 treatment alone, a cell count of treated and control dishes was performed that shows a decrease in the number of cells after 24hrs of treatment for all conditions compared to control. However, the decrease with ABT-263 treatment, 66% of control, was not significant while treatment with TAK-228 and the combination caused a significant decrease in cell counts, down to 44% of control in each condition (p<0.01) [Fig. 3(a)]. Viability was further confirmed with a WST-1 plate-based assay, which relies on the cleavage of WST-1 to formazan by mitochondrial dehydrogenases. These measurements agree with the cell count measurements, as ABT-263 treatment alone causes no significant decrease in cell viability compared to control while TAK-228 and combination treatments cause a significant decrease in the percent of viable cells, down to 66% of control with both treatments [Fig. 3(b)]. Therefore, cell viability is not significantly affected by ABT-263 treatment alone in SW48 cells confirming our prior results.^26^ Cell proliferation and cell death are related to cell viability, so we performed immunofluorescence staining for Ki67 and cleaved caspase 3 (CC3). The percent positive cells per FOV shows a small but non-significant decrease in the percent of cells proliferating in all treatment conditions compared to control [Fig. 3(c)]. However, the CC3 stain shows a small but significant increase in CC3 positive cells with ABT-263 and combination treatments, with 3% CC3 positive cells in the control condition to 5% to 6% CC3 positive cells in the ABT-263 and combination conditions [Fig. 3(d)]. Though there is a small increase in the percent of cells that are CC3 positive, an increase from 3% to 6% is not likely to account for the significant changes in the ORR and energetic state of the SW48 cells. These measurements [Figs. 3(a)–3(d)] confirm that cell viability is not significantly affected by ABT-263 treatment alone in SW48 cells, motivating further investigation into cell states that are known to be controlled by Bcl-2 family proteins including autophagy and senescence.

Autophagy was measured with the Cyto-ID plate-based assay, which measures the uptake of a green dye into the autophagosomes of cells as they go through autophagy.^54^ Autophagy is dynamic and difficult to measure, so pre-treatment with chloroquine, an inhibitor of autophagosome degradation, was used as a standard method to detect the signal in this assay.^55^^–^^57^ Chloroquine blocks the ability of the cell to degrade autophagosomes and thereby increases the green signal that is detected by the assay.^56^^,^^58^ Without the addition of chloroquine, there was a low level of fluorescence detected in all conditions, as the autophagosomes likely formed and were then lysed prior to plate reading, and only the combination treatment showed a small but significant increase from control [Fig. 3(e)]. For this assay, rapamycin, an mTORC1/2 inhibitor known to induce autophagy was used as a positive control. However, without chloroquine treatment to stop the degradation of autophagosomes, rapamycin showed no difference compared to control [Fig. 3(e)], which confirms that the signal was below reliable detection levels. After the addition of chloroquine, autophagosomes remain in the cells allowing for more accurate interpretation of autophagy. In this condition the rapamycin positive control showed the expected significant increase in autophagosome fluorescence compared to control. Additionally, in this condition there was increased autophagy with TAK-228 and combination treatment [Fig. 3(e)]. This is expected as TAK-228 is a powerful mTORC1/2 inhibitor, like the positive control rapamycin, and should cause an increase in autophagy. However, ABT-263 treatment alone did not cause an increase in the autophagosome fluorescence compared to control, indicating that the increase in ORR and energetic state is not due to an increase in autophagy.

Finally, we investigated whether treatments induced cell senescence with a β-galactosidase stain (X-gal). Control cells were 28% positive for senescence [Fig. 3(f)], which is comparable to senescence levels previously measured in control SW48 cells.^59^^,^^60^ There was a slight non-significant decrease in the percent of senescent cells in both the TAK-228 and combination treatment conditions. Notably, there was a significant increase in the percent of cells positive for senescence after ABT-263 treatment alone, up to 40% [Fig. 3(f)]. This increase in the percent of cells positive for senescence indicates that there may be a senescence driven change in the metabolism of the SW48 cells after ABT-263 treatment. This is supported by prior work which has shown that senescence increases the OCR and ECAR in a variety of cell types.^61^^–^^64^ Overall, these cell state measurements (Fig. 3) show that ABT-263 does not change cell viability or induce autophagy but likely induces a senescent phenotype in SW48 cells.

## 4 Conclusions

**Fig. 4:**
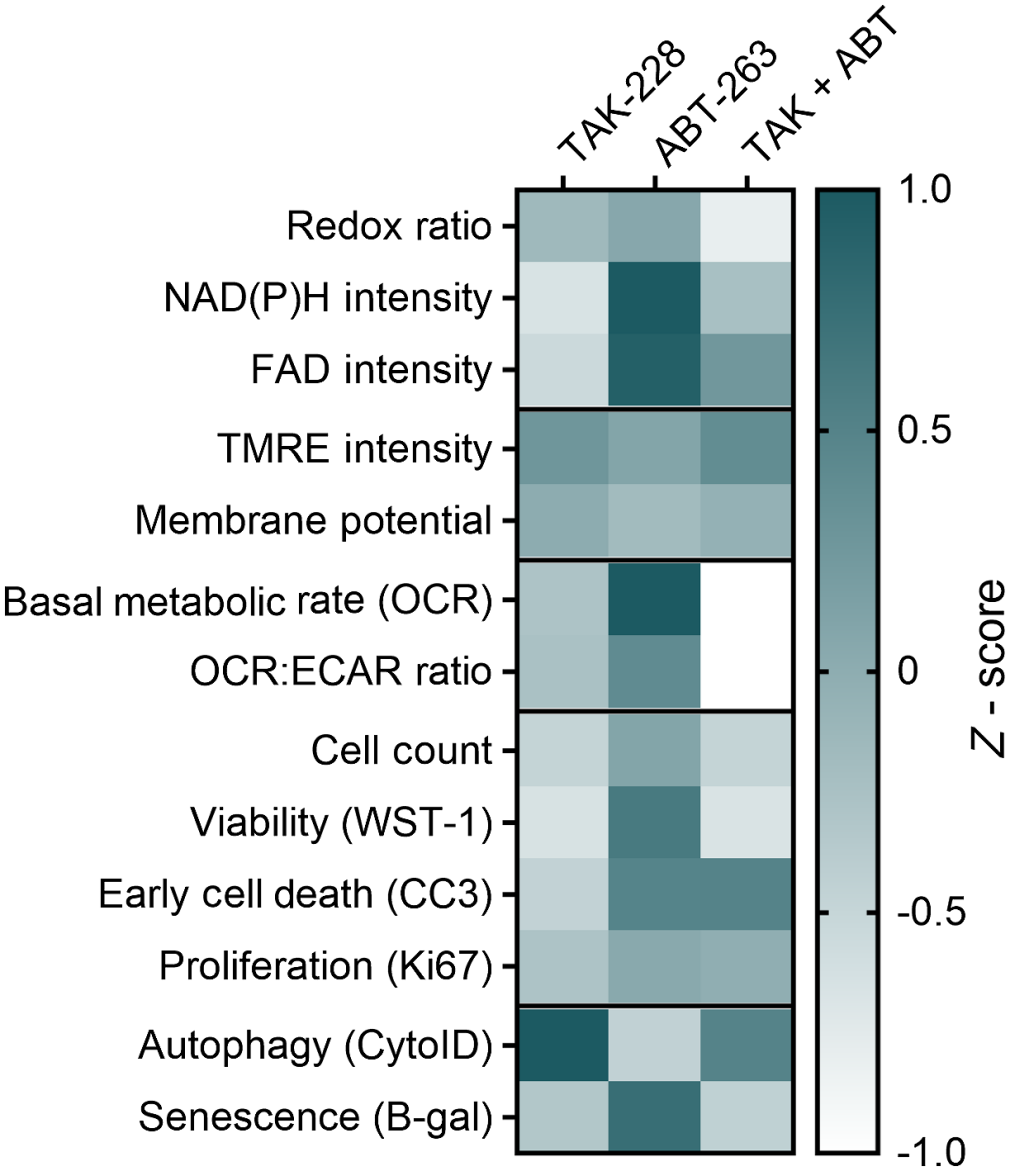
Summary of the data presented in this article. Heatmap of the z-score $\left[z\text{-score}=(\mu_{\text{treatment}}-\mu_{\text{control}})/\sigma_{\text{control}}\right]$ for each treatment group compared to control for all measurements taken in SW48 cells in this study [redox ratio is the intensity of NAD(P)H divided by FAD].

Bcl-2 family proteins control many cellular processes including MOMP, autophagy, senescence, inflammation, bioenergetic metabolism, and redox homeostasis, which can all affect cellular state.^30^ This family of proteins plays an important role in a complex series of cell health and cell state pathways that directly affect the ORR.^13^^,^^26^^,^^30^^,^^31^^,^^65^^,^^66^ Typically, a decrease in the ORR is an early indicator of treatment response.^19^^,^^46^ However, the decrease in ORR with ABT-263 treatment is driven by an increased energetic state, reflected by increased NAD(P)H and FAD intensities and OCR (Seahorse measurements), but is not accompanied by significant changes in viability or autophagy (Fig. 4). Published work has shown that senescence increases OCR and ECAR in a variety of cell types.^61^^–^^64^ This increase correlates with an increase in the concentrations of NAD(P)H and FAD as measured by optical redox imaging. No prior studies have directly related the ORR to cellular senescence. However, previous ORR imaging of young and old mouse articular cartilage supports this result as both NAD(P)H and FAD intensities increased with cartilage age^67^ and senescence is also known to increase with age.^68^

Interestingly, the increased energetic state with ABT-263 treatment alone is not seen when ABT-263 is used in combination with mTORC 1/2 inhibition (TAK-228). The combination treatment causes a reduction in cell viability, increase in autophagy, and no change in senescence compared to control (Fig. 4). This suggests that the senescent phenotype of ABT-263 treatment alone is not the cause of the decreased ORR in the combination treated cells and supports the synergistic effect of ABT-263 and TAK-228 combination treatment where decreased cell viability and increased autophagy are the primary factors influencing the ORR. Prior work supports this synergistic effect of Bcl-2 inhibition and mTORC 1/2 inhibition in combination.^33^^,^^35^ Additional dissection of this potential treatment synergy is outside the scope of this article. Furthermore, it is important to note that ABT-263 inhibits multiple Bcl-2 family proteins, including Bcl-2, Bcl-xl, and Bcl-w proteins, which each play a different role in cellular signaling.^28^ Overall, these studies show that the ORR is sensitive to metabolic changes in mitochondrial polarization and cell energetics, which can change independently from cell state. Furthermore, this work emphasizes the importance of understanding the metabolic effects of treatments when using the ORR to investigate treatment response, especially in the case of senesence. Here, we find that ABT-263 treatment decreases the ORR, which is commonly associated with treatment response, but this decrease in ORR with ABT-263 treatment is due to a cell state shift toward senescence rather than a change in cell viability.

## Code, Data, and Materials Availability

This article does not have associated code. The data presented in this study are available on request from the corresponding author.
